# Hypertensive Retinopathy Is Associated With Worse Cognitive Function in Women: The SWAN Study

**DOI:** 10.1093/geroni/igab046.046

**Published:** 2021-12-17

**Authors:** Sayoko Moroi, Michelle Hood, Carrie Karvonen Gutierrez, Joshua Ehrlich, Brenda Gillespie, Sarah Dougherty Wood, David Musch

**Affiliations:** 1 The Ohio State University, Columbus, Ohio, United States; 2 University of Michigan, Ann Arbor, Michigan, United States; 3 University of Michigan, University of Michigan, Michigan, United States

## Abstract

Based on the 2018 American Academy of Neurology guideline, the prevalence of mild cognitive impairment (CI) increases from 6.7% at 60-64 years to 25.2% at 80-84 years. There is interest to identify potential biomarkers in the retina for CI and dementia. The aims of this analysis was to test whether hypertensive retinopathy (HR) was associated with cognitive function using data from the Study of Women’s Health Across the Nation (SWAN), Michigan cohort. SWAN, launched in 1996/97, is a longitudinal study of women traversing midlife and into late adulthood. Starting in 2000, cognitive function tests were administered: East Boston Memory Test immediate (EBMTi) and 10-minute delay (EBMTd) for verbal episodic memory; digit span backwards (DSB) for working memory; and symbol digits modalities test (SDMT) for perception speed, motor speed, and visual scanning. Z-scores were calculated for EBMTi, EBMTd, DSB and SDMT and averaged at follow-up visit 15 (2015/16). Eye exams were performed on 255 women (66 + 2.6 years) at follow-up visit 16 (2016/17). HR was based on presence/absence of arteriovenous nicking. Logistic regression showed a statistically significant association of lower average cognitive Z-score with HR (p-value 0.03, beta=-0.21 [95% confidence interval: -0.40 to -0.02]) adjusting for measured hypertension or anti-hypertensive drugs, years of measured hypertension, race, education, and age. Preliminary results indicate that HR is associated with lower cognitive test scores in women in their 60s-70s. This association between a simple ophthalmic exam finding of systemic hypertension, i.e., arteriovenous nicking, and lower cognitive function is consistent with a cerebrovascular mechanism of CI.

